# Expression of K_2P_5.1 potassium channels on CD4^+ ^T lymphocytes correlates with disease activity in rheumatoid arthritis patients

**DOI:** 10.1186/ar3245

**Published:** 2011-02-11

**Authors:** Stefan Bittner, Nicole Bobak, Martin Feuchtenberger, Alexander M Herrmann, Kerstin Göbel, Raimund W Kinne, Anker J Hansen, Thomas Budde, Christoph Kleinschnitz, Oliver Frey, Hans-Peter Tony, Heinz Wiendl, Sven G Meuth

**Affiliations:** 1 Department of Neurology, University of Wuerzburg, Josef-Schneider-Str. 11, Wuerzburg, 97080, Germany; 2 Department of Neurology - Inflammatory disorders of the nervous system and neurooncology, University of Muenster, Domagkstr. 13, Muenster, 48149, Germany; 3 Department of Medicine II - Rheumatology and Clinical Immunology, Josef-Schneider-Str. 2, University of Wuerzburg, Wuerzburg, 97080, Germany; 4 Department of Orthopedics - Experimental Rheumatology Unit, Friedrich Schiller University Jena, Klosterlausnitzer Str. 81, Eisenberg, 07607, Germany; 5 Biopharmaceuticals Research, Novo Nordisk A/S, Novo Nordisk Park, Malov, 2760 Denmark; 6 Institute of Physiology I, University of Muenster, Robert-Koch-Str. 27a, Muenster, 48149, Germany; 7 Institute of Immunology, University Hospital Jena, Leutragraben 3, Jena, 07743, Germany

## Abstract

**Introduction:**

CD4^+ ^T cells express K_2P_5.1 (TWIK-related acid-sensitive potassium channel 2 (TASK2); KCNK5), a member of the two-pore domain potassium channel family, which has been shown to influence T cell effector functions. Recently, it was shown that K_2P_5.1 is upregulated upon (autoimmune) T cell stimulation. The aim of this study was to correlate expression levels of K_2P_5.1 on T cells from patients with rheumatoid arthritis (RA) to disease activity in these patients.

**Methods:**

Expression levels of K_2P_5.1 were measured by RT-PCR in the peripheral blood of 58 patients with RA and correlated with disease activity parameters (C-reactive protein levels, erythrocyte sedimentation rates, disease activity score (DAS28) scores). Twenty patients undergoing therapy change were followed-up for six months. Additionally, synovial fluid and synovial biopsies were investigated for T lymphocytes expressing K_2P_5.1.

**Results:**

K_2P_5.1 expression levels in CD4^+ ^T cells show a strong correlation to DAS28 scores in RA patients. Similar correlations were found for serological inflammatory parameters (erythrocyte sedimentation rate, C-reactive protein). In addition, K_2P_5.1 expression levels of synovial fluid-derived T cells are higher compared to peripheral blood T cells. Prospective data in individual patients show a parallel behaviour of K_2P_5.1 expression to disease activity parameters during a longitudinal follow-up for six months.

**Conclusions:**

Disease activity in RA patients correlates strongly with K_2P_5.1 expression levels in CD4^+ ^T lymphocytes in the peripheral blood in cross-sectional as well as in longitudinal observations. Further studies are needed to investigate the exact pathophysiological mechanisms and to evaluate the possible use of K_2P_5.1 as a potential biomarker for disease activity and differential diagnosis.

## Introduction

Rheumatoid arthritis (RA) is a chronic inflammatory disease which is characterized by pain, swelling and progressive destruction of multiple joints. The systemic nature of RA causes, next to loss of joint function, substantially decreased quality of life and increased mortality of patients. Current treatments are mainly based on immunosuppressive disease-modifying antirheumatic drugs, among them the rapidly expanding family of biologic agents [1]. Close monitoring of disease activity is mandatory for the evaluation of treatment efficacy as a substantial percentage of patients do not respond adequately to first-line therapy. In these cases, as well as in patients with disease exacerbations, a change in treatment strategy is required [2]. Monitoring of disease activity includes patient history, clinical examination, blood values (C-reactive protein (CRP) levels and erythrocyte sedimentation rate (ESR)) and composite scores such as the widely used disease activity score (DAS28). The DAS28 score includes the number of swollen and painful joints, the ESR rate and the patient's subjective evaluation on a visual analogy scale (VAS) [3,4].

The potassium channel K_2P_5.1 (TWIK-related acid-sensitive potassium channel 2 (TASK2); KCNK5) belongs to the family of two-pore domain potassium channels (K_2P _channels) which has recently been shown to be expressed on T lymphocytes [5,6]. K_2P_5.1 is important for T cell functions such as proliferation or cytokine production [7] as it is hypothesized that the counterbalancing efflux of potassium channels is mandatory for a longer lasting elevation of the intracellular Ca^2+ ^levels during T cell stimulation [7]. Moreover, chronic repetitive stimulation leads to an upregulation of K_2P_5.1 channel expression whereas pharmacological blockade or siRNA-induced gene silencing of K_2P_5.1 results in a reduction of T cell effector functions. It has additionally been shown that expression levels of this channel are strongly increased on T lymphocytes from the peripheral blood from clinically active relapsing-remitting multiple sclerosis (MS) patients. Interestingly, expression and MS-specific upregulation were found predominantly on CD8^+ ^T cells rather than on CD4^+ ^T cells which may be due to a disease-specific pathogenic role of cytotoxic T lymphocytes. Expression of K_2P_5.1 was even higher on cerebrospinal fluid (CSF)-derived T lymphocytes than in the peripheral blood and K_2P_5.1-positive T lymphocytes can be found within inflammatory lesions from MS patients. So far, it was not known whether these findings are MS-specific or can be similarly found in other autoimmune disorders. CD4^+ ^T helper cells play an important role in the pathogenesis of RA. This is suggested by its association with certain MHC II loci, especially HLA-DRB1, and PTPN22, which is relevant for T cell function [8]. The therapeutic effects of blockade of T cell costimulation by abatacept provides more direct evidence [9].

Therefore, we investigated the correlation of K_2P_5.1 expression levels on T lymphocytes from RA with different disease activity parameters. The influence of different therapies was taken into account as they might potentially influence K_2P_5.1 expression. Finally, a longitudinal study was conducted in a subset of patients who underwent therapy change due to disease exacerbation and these patients were followed up for six months.

## Materials and methods

### Material from RA patients

T lymphocytes were isolated from the peripheral blood of 73 RA patients (cross-sectional-study: 58 patients, longitudinal study: 20 patients, included in both studies: 5 patients) and 10 age- and sex-matched healthy donors. The patients were seen between April 2009 and August 2010 in the outpatient RA center at the University of Wuerzburg. We differentiated the patients according to EULAR criteria in patients in remission (DAS28 ≤2.6), patients with low disease activity (2.6 <DAS28 ≤3.2), with moderate disease activity (3.2 <DAS28 ≤5.1) and high disease activity (DAS28 >5.1). The DAS28 specifies the disease activity measuring the number of tender and swollen joints, the ESR and VAS of general health. Conventional therapy with disease modifying anti-rheumatic drugs (DMARDs) included leflunomide, hydroxychloroquine, sulfasalazine, methotrexate and glucocorticoids. Treatment with biologic agents included tumor necrosis factor alpha (TNFα) blockers (etanercept, adalimumab, infliximab and certolizumab) and anti-CD20 treatment (rituximab). Patients receiving interleukin (IL)-6 receptor blocking antibodies (tocilizumab) were excluded from the primary analysis because of its direct influence on CRP and ESR levels. However, they were included into the longitudinal study due to their influence on CRP and ESR values. In additional sets of experiments, the synovial fluid of patients who underwent joint punctures for diagnostic or therapeutic purposes was investigated. See Table 1 for details on the RA patients. All patients gave informed consent in accordance with the Declaration of Helsinki and a protocol approved by the Ethics Committee of the University of Wuerzburg Medical School (No. 109/10).

**Table 1:** Characteristics of RA patients

Patients (male/female)	19/54
Age	56.6 (23 to 79)
DAS28	3.59 (1.10 to 7.24)
ESR	19.3 (1.0 to 69.0)
CRP	1.06 (0.02 to 4.68)

All of the probands had been diagnosed with RA by a board-certified rheumatologist. Values are depicted as mean and range. Abbrevations: CRP, C-reactive protein; DAS28, disease activity score in 28 joints; ESR, erythrocyte sedimentation rate.

### Cell isolation

Peripheral blood mononuclear cells (PBMCs) from RA patients and healthy donors were isolated out of fresh blood samples by density gradient centrifugation using a lymphocyte separation medium (PAA Laboratories, Pasching, Austria). CD4^+ ^and CD8^+ ^T cells were separated by magnetic cell sorting (MACS) according to the manufacturer's instruction (Miltenyi, Bergisch Gladbach, Germany) and purity was >95%. Direct cell isolation had no effect on K_2P_5.1 expression when compared to indirect cell isolation (data not shown). Synovial fluid from patients undergoing joint puncture for therapeutic or diagnostic purposes was processed accordingly.

### Real-time RT-PCR

For analysis of K_2P_5.1-mRNA expression, RNA was purified using Trizol reagent (Invitrogen, Carlsbad, CA, USA) and cDNA synthesis was performed using a standard protocol with random hexamer primers (all reagents were purchased from Applied Biosystems, Foster City, CA, USA). This cDNA was used in a RT-PCR assay with specific primers for KCNK5 (Hs00186652_m1; FAM-labeled; Applied Biosystems) and endogen control primers for 18sRNA (Hs_4319413E; VIC-labeled; Applied Biosystems). Real time RT-PCR was performed according to the manufacturer's protocol.

In one set of experiments cultured CD4^+ ^T cells from healthy donors were treated with methotrexate (Medac, Hamburg, Germany), etanercept (Wyeth Europa Ltd., Maidenhead, Berkshire, UK), adalinumab (Abbott Laboratories Ltd., Maidenhead, Berkshire, UK), certolizumab (Ucb S.A. Brussels, Belgium), tocilizumab (Roche, Welwyn Garden City, UK) or hydroxychloroquine (Sanofi-Aventis, Frankfurt am Main, Germany) over 24 hours before PCR analysis.

### Western blotting

Whole cell lysates from unstimulated and CD3/CD28-bead stimulated MACS-isolated CD4^+ ^T lymphocytes were analyzed as described previously [6] using rabbit anti-K_2P_5.1 and HRP-donkey anti-rabbit (Amersham, Freiburg, Germany). HRP was inactivated with 2% NaN_3 _and blots were stained with β-actin antibody for protein loading control. Quantification of Western blot results was performed using Image J.

### Flow cytometry analysis

The following antibodies were used: rabbit anti-K_2P_5.1 (Sigma, St. Louis, MO, USA) and goat anti-rabbit Cy2 (Dianova, Hamburg, Germany; intracellular staining), CD4-FITC (RPA-T4), CD69-PerCP (BD Pharmingen, Franklin Lakes, NJ, USA), and CD25-PE (Miltenyi). Flow cytometry was done using a FACSCalibur system (BD Bioscience, Heidelberg, Germany) and CellQuest Pro Software (BD Bioscience).

### Immunfluorescence staining

Immunfluorescence staining was performed on human synovial tissue sections (n = 5). For double labelling, slices were postfixated in 4% paraformaldehyde (PFA) and incubated in blocking solution. Slices were then incubated consecutively with anti-CD3 (1:100, Dako, Glostrup, Denmark) and TASK2 (Sigma). Secondary antibodies were Alexa goat anti-mouse Fluor 488 and goat anti-rabbit Cy3 (Dianova, Hamburg, Germany).

### Statistical analysis

All results are presented as mean ± standard error of measure (SEM). Statistical analysis was performed using a modified Student's *t*-test [10] in case of normally distributed data, or a Mann-Whitney test otherwise. Spearman's rank correlation was used for correlation analysis. *P*-values < 0.05 were considered statistically significant.

## Results

**Figure 1:**
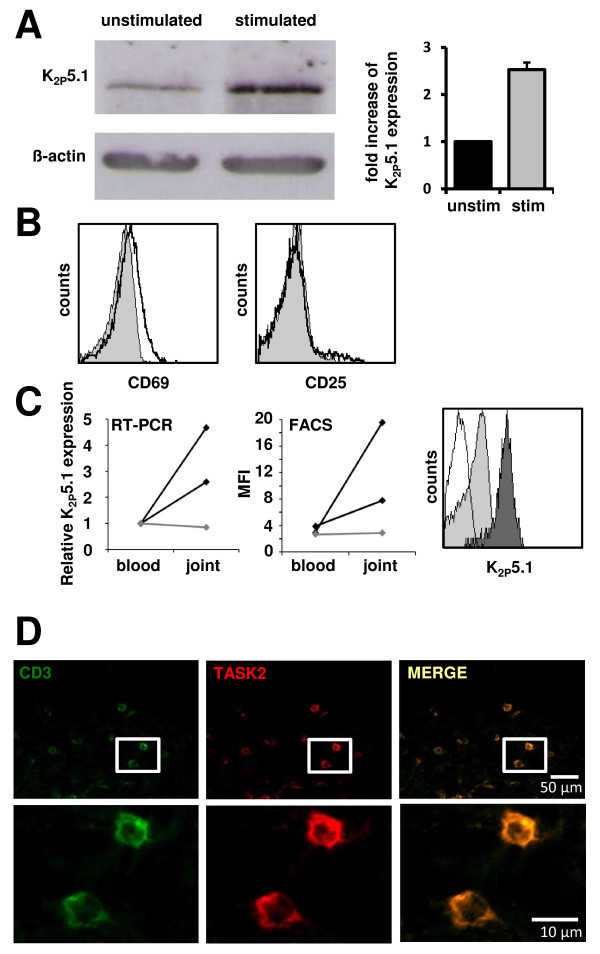
**Comparison of CD4^+ ^T lymphocytes derived from peripheral blood and synovial fluid**. **A) **Western blotting of unstimulated and stimulated CD4^+ ^T cells for K_2P_5.1 and β-actin as loading control (left panel). Quantification of three independent Western blotting experiments (right panel). **B) **Exemplary flow cytometry stainings for the activation markers CD69 (left panel) and CD25 (right panel) are shown for cells derived from the peripheral blood (grey) and synovial fluid (black). **C) **K_2P_5.1 expression levels on CD4^+ ^cells were compared by RT-PCR (left panel) and flow cytometry staining (middle panel). Black lines: RA; grey lines: reactive arthritis. One representative example for RA is depicted on the right side (white: isotyp, grey: peripheral blood, black: synovial fluid). **D) **Immunohistochemical staining of human synovial tissue sections for CD3 (left panel), TASK2 (middle panel) and overlay (right panel).

The potassium channel K_2P_5.1 has been previously shown to regulate T cell function *in vitro *and *in vivo*. In a first set of experiments, freshly isolated CD4^+ ^T cells from healthy donors were either left unstimulated or stimulated with CD3/CD28 beads for two days. A clear upregulation of K_2P_5.1 could be shown on protein level (one representative example is shown in Figure 1A, left panel; quantification of three independent experiments - right panel). In MS, putatively pathogenic T cells derived from the CSF - reflecting the site of inflammation - showed significantly higher levels of K_2P_5.1 than T cells from the peripheral blood of the same patients [7]. Based on these findings, we next analyzed the K_2P_5.1 expression on activated CD4^+ ^T lymphocytes out of the synovial fluid of RA patients. Synovial fluid derived T lymphocytes showed a slight upregulation of the activation marker CD69, but not of CD25 when compared to cells from the peripheral blood (Figure 1B). In patients with RA, K_2P_5.1 is upregulated both on RNA level (Figure 1C, left panel, black lines) and on protein level (Figure 1C, middle panel, black lines) as assessed by real time RT-PCR and flow cytometry, respectively. A representative flow cytometry staining can be found in Figure 1C, right panel. In contrast, no difference could be observed in one patient with reactive arthritis (Figure 1C, left and middle panel, grey lines). These findings point towards a shared pathophysiological motif of both T cell-mediated disorders - namely MS (CD8^+ ^T cells) and RA (CD4^+ ^T cells). Finally, TASK2 expressing T cells could be identified immunohistochemically within human synovial tissue sections. Exemplary costainings for the T cell marker CD3 and TASK2 can be found in Figure 1D.

**Figure 2:**
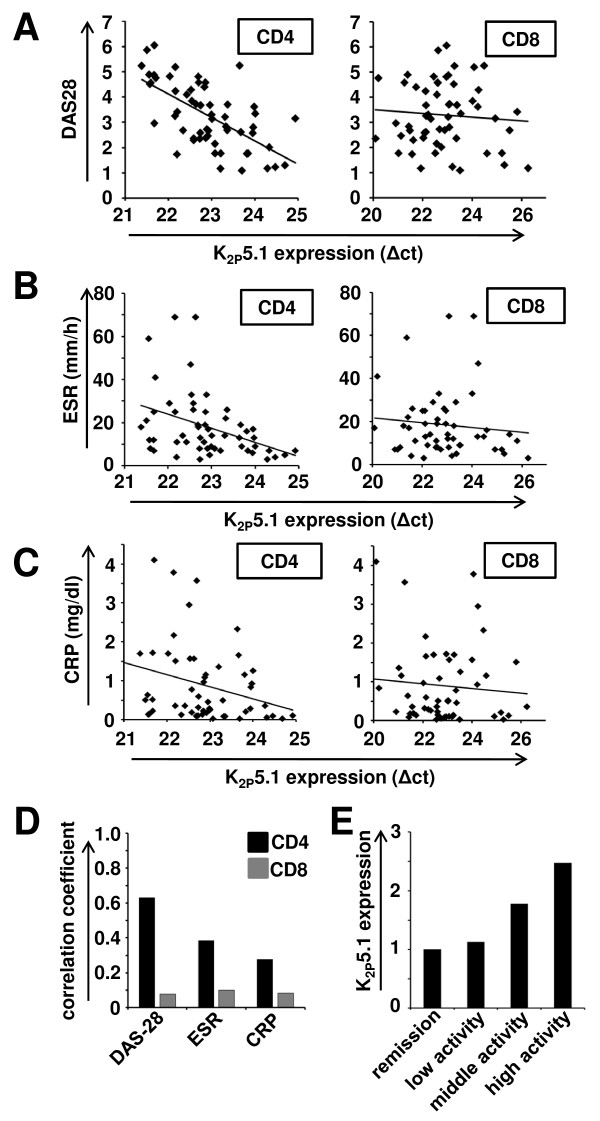
**K_2P_5.1 expression on CD4^+ ^T lymphocytes correlates with disease activity parameters**. **A) **Expression levels of K_2P_5.1 (Δct values) and DAS28 scores are shown for 58 individual patients for CD4^+ ^(left side) and CD8^+ ^(right side) T lymphocytes. Note that the x-axis (Δct values) is logarithmic scale and that lower Δct values mean higher gene expression. **B) **Δct values for K_2P_5.1 and ESR rates (mm/h) are depicted for CD4^+ ^(left side) and CD8^+ ^(right side) T lymphocytes. **C)** Δct values for K_2P_5.1 and CRP levels (mg/dl) are shown for CD4^+ ^(left side) and CD8^+ ^(right side) T lymphocytes. **D)** The correlation coefficient for K_2P_5.1 and DAS28, ESR or CRP is shown for CD4^+ ^and CD8^+ ^T lymphocytes. **E)** K_2P_5.1 expression levels on CD4^+ ^T cells are shown on clinically defined patient subgroups as stated in the Material and Methods section.

Activity of CD4^+ ^and CD8^+ ^T lymphocytes critically depend on constitutively expressed K_2P_5.1 potassium channels. Chronic stimulation as it may occur under autoimmune conditions leads to an upregulation of these channels on RNA and protein level. In a first set of experiments, MACS-isolated T cells from the peripheral blood of 58 RA patients were analyzed for expression of K_2P_5.1 by RT-PCR. We compared the correlation of K_2P_5.1 channel expression on both CD4^+ ^and CD8^+ ^T lymphocytes with different disease activity parameters. We found a positive correlation between K_2P_5.1 expression levels measured as Δct values and DAS28 on CD4^+ ^T lymphocytes (R = 0.63; Figure 2A left panel; Figure 2D). Moreover, a weaker correlation could be found for ESR (R = 0.39; Figure 2B left panel; Figure 2D) and CRP levels in the peripheral blood (R = 0.28; Figure 2C left panel; Figure 2D). In contrast, K_2P_5.1 expression on CD8^+ ^T lymphocytes and three disease activity parameters were found to be only weakly correlated (Figure 2A-D). In summary, K_2P_5.1 expression on CD4^+ ^T lymphocytes of RA patients seems to be positively correlated with the disease severity (Figure 2E). Hence, K_2P_5.1 expression levels are strongly elevated in patient subgroups with high disease activity (Figure 2E). These results were corroborated on protein level in a small cohort of patients (n = 5, see Supplementary Figure S1 in Additional file 1). As a note of caution, it should be mentioned that all RA patients received disease-modifying therapies, which were divided into three classes due to their mode of action (conventional, black diamonds; TNFα inhibitors, grey diamonds; rituximab, white diamonds; Supplementary Figure S2A-B in Additional file 2). The correlation coefficients for these subgroups are shown in Supplementary Figure S2C in Additional file 2 (conventional: n = 21, R = 0.61; TNFα inhibitors: n = 27, R = 0.49; rituximab: n = 10, R = 0.91). It may be speculated that the high positive correlation which was found for rituximab-treated patients may at least partly be due to a higher disease activity in this patient subgroup (conventional: DAS28 = 2.92; TNFα inhibitors: DAS28 = 3.30; rituximab: DAS28 = 3.98). To further support these results we analyzed K2P5.1 expression in naïve and stimulated CD4^+ ^T cells from healthy individuals after 24 hours *in vitro *treatment with methotrexate, etanercept, adalinumab, certolizumab, tocilizumab and hydroxychloroquine. No significant upregulation of K2P5.1 was observed in treated T cells compared to untreated controls (n = 5; see Table 2).

**Table 2:** *In vitro *effects of methotrexate, etanercept, adalinumab, certolizumab, tocilizumab and hydroxychloroquine on K2P5.1 expression levels

Substance	Concentration (μm)	CD4 T cells, unstimulated	CD4 T cells, stimulated
control	--	1.00	1.00
methotrexate	20 μM	1.08 ± 0.18	0.93 ± 0.08
etanercept	10 μg/ml	0.97 ± 0.07	1.01 ± 0.04
adalinumab	10 μg/ml	0.96 ± 0.14	1.12 ± 0.05
certolizumab	10 μg/ml	0.99 ± 0.09	1.14 ± 0.08
tocilizumab	10 μg/ml	1.12 ± 0.22	1.24 ± 0.15
hydroxychloroquine	2 μg/ml	0.82 ± 0.3	0.84 ± 0.35

Normalized values of K2P5.1 expression are depicted as mean ± SE.

**Figure 3:**
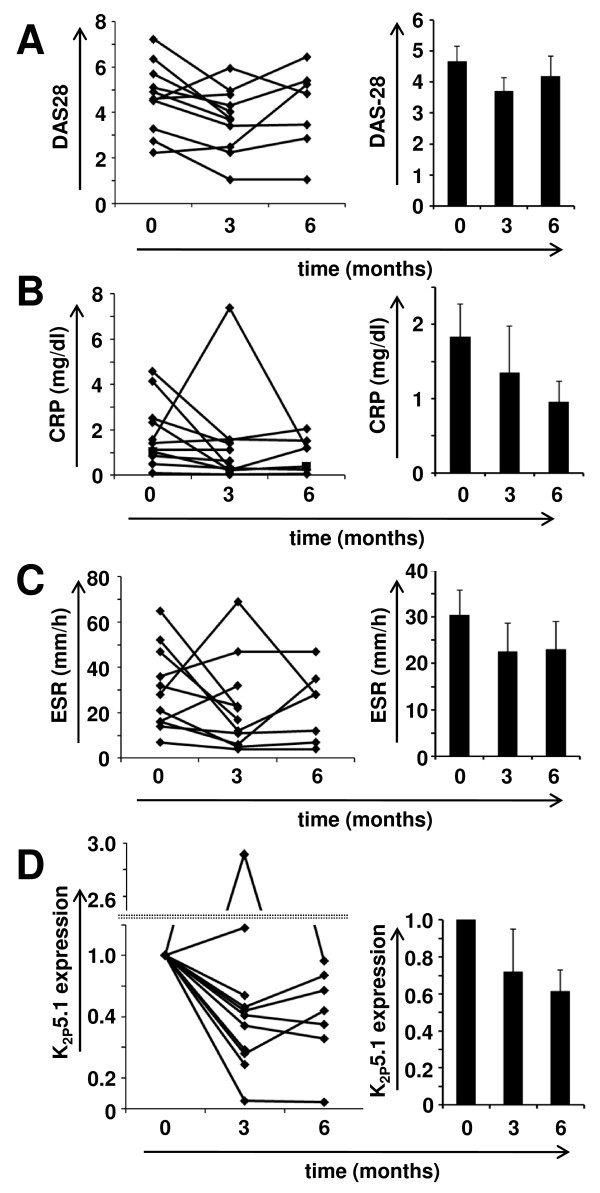
**A longitudinal follow-up study of RA patients undergoing therapy change**. Comparison of **A) **DAS28, **B) **CRP values, **C) **ESR values and **D) **relative K_2P_5.1 expression levels at therapy change (t = 0), after three months (t = 3) and six months (t = 6) of follow-up. Left panel shows individual values for all patients while mean values ± SEM are depicted on the right side. Note that the y-axis in D) is split for better clarity.

In a next set of experiments a longitudinal follow-up study was initiated including patients who underwent therapy escalation from conventional therapy to biological or from one biological to another. According to these criteria, 11 patients were recruited and followed up for three to six months. Most patients (n = 8) showed at Month 3 a reduction in the DAS28 score (Figure 3A, left panel). Mean DAS28 scores are shown on the right (t = 0 months: 4.66 ± 0.50; t = 3 months: 3.70 ± 0.42; t = 6 months: 4.18 ± 0.70). Comparable results could be found for CRP (Figure 3B) and ESR levels in the peripheral blood (Figure 3C) which decreased from 1.84 mg/dl to 0.95 mg/dl and from 30.4 mm/h to 22.9 mm/h, respectively. K_2P_5.1 expression levels decreased at Month 3 in 9 out of 11 patients (Figure 3D). It should be noted that two patients who had an increase of K_2P_5.1 expression at Month 3 showed a parallel increase of DAS28. In the entire group the relative expression reduction of K_2P_5.1 was 28% and 47% at three and six months, respectively. In summary, this prospective study demonstrates a strong longitudinal correlation of clinical disease activity parameters with K_2P_5.1 expression levels of peripheral CD4^+ ^T cells in individual patients.

**Figure 4:**
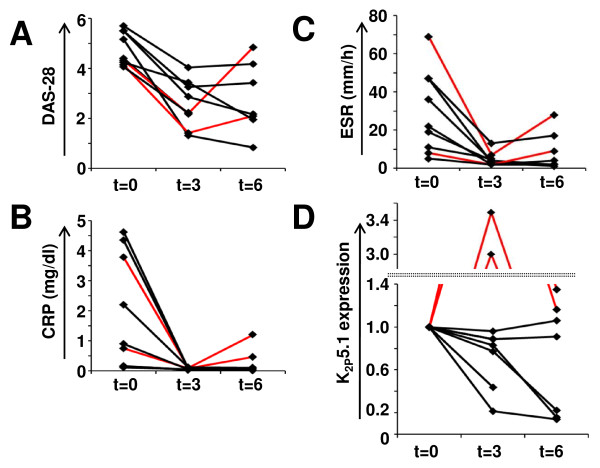
**A longitudinal study of RA patients receiving tocilizumab**. **A) **DAS28 **B) **CRP values, **C) **ESR values and **D) **relative K_2P_5.1 expression levels are compared for patients with therapy switch to tocilizumab over six months. Note that the y-axis in D) is split for better clarity and that two patients are marked in red.

In an additional set of experiments, nine patients with a therapeutic switch to tocilizumab were followed up for six months as well (Figure 4). By interfering with IL-6 signaling, tocilizumab is known to have inhibitory effects on inflammatory markers such as CRP, serum amyloid A or ESR [11]. These effects of tocilizumab may be independent of its therapeutic effect in RA patients and it has been doubted whether classical surrogate markers (for example, ESR, CRP) are suitable for measuring therapy efficacy in tocilizumab-treated RA patients [12]. Indeed, we found a drastic decrease in ESR (29.3 ± 0.8 to 4.7 ± 1.5 mm/h) and CRP (1.89 ± 0.78 to 0.06 ± 0.01 mg/dl) values three months after tocilizumab initiation. At three months, all patients showed an initial decrease in DAS28, ESR and CRP values, whereas K_2P_5.1 levels were clearly downregulated in four and strongly upregulated in two patients to 3.0 and 3.5, respectively (marked in red throughout Figure 4A-D). Remarkably, in both patients K_2P_5.1 upregulation preceded a clinical relapse.

## Discussion

Disease activity in RA patients was found to correlate strongly with K_2P_5.1 expression levels in CD4^+ ^T lymphocytes in the peripheral blood in a cross-sectional study in 58 patients. Furthermore, longitudinal observations in individual patients showed comparable changes in all disease surrogate markers with K_2P_5.1 expression. It seems plausible that K_2P_5.1 expression reflects the activation status of chronically stimulated autoimmune CD4^+ ^T lymphocytes.

However, a number of questions remain to be addressed which are beyond the focus of this initial study, for example: Can K_2P_5.1 expression serve as a biomarker for disease activity in RA? Current serum markers (CRP and ESR levels) which are also part of composite scores like DAS28 or DAS28-CRP reflect rather the level of systemic inflammation than the specific activation status of pathogenic immune cells. Moreover, their use seems to be limited especially in the case of tocilizumab which inhibits the systemic acute phase reaction at least in part independently from its well-proven anti-rheumatic effects [13]. This problem has, for example, been addressed by Matsui *et al. *who evaluated neutrophil CD64 as a biomarker for otherwise masked infection under tocilizumab therapy [14]. We observed that in contrast to our cross-sectional study and our longitudinal study with other medications, K_2P_5.1 expression changes behaved differently in tocilizumab-treated patients. Two out of nine patients even showed an opposite upregulation of K_2P_5.1 at three months preceding a relapse about three months later. At the present, it seems too early and patient numbers are too low to state that K_2P_5.1 upregulation generally precedes clinical relapses in RA, whether it reveals otherwise masked clinical developments under tolizumab therapy or whether its validity is limited under these circumstances. Therefore, in light of the current results further patient studies need to assess the prognostic value, time course and clinical validity of monitoring K_2P_5.1 expression in RA patients. Furthermore, it has to be proven whether K_2P_5.1 can be used for differential diagnosis.

Additionally, research efforts are needed concerning the mechanisms underlying K_2P_5.1 function and regulation in the pathophysiology of RA. Especially the use of animal models for RA may help to provide insight in this context.

## Conclusions

We show here for the first time a correlation of K_2P_5.1 expression levels in CD4^+ ^T lymphocytes and disease activity in patients suffering from RA. Since other studies already showed a functional role of K_2P_5.1 for T cell effector function this member of the two-pore domain potassium channel family might represent an interesting molecular target for diagnostic and/or therapeutic applications. However, further studies from independent cohorts are warranted to confirm and extend the presented findings. Furthermore, the use of animal models for RA might help to shed more light on the functional role of K_2P_5.1 in RA pathogenesis.

## Abbreviations

CRP: C-reactive protein; CSF: cerebrospinal fluid; DAS28: disease activity score of 28 joints; DMARDs: disease modifying anti-rheumatic drugs; ESR: erythrocyte sedimentation rate; MACS: magnetic cell sorting; MS: Multiple Sclerosis; PBMCs: peripheral blood mononuclear cells; PFA: paraformaldehyde; RA: rheumatoid arthritis; TASK2: TWIK-related acid-sensitive potassium channel 2; VAS: visual analogy scale.

## Competing interests

The authors declare no conflict of interest. AJH, SB, NB, HW and SGM collaborate in a project on K_2P_5.1-biology in inflammatory conditions.

## Authors' contributions

SB, NB and AHM isolated cells and performed RT-PCR. SB performed flow cytometry and NB was responsible for Western blotting. MF recruited the RA patients and assessed clinical data. KG and AJH performed immunohistochemical stainings on synovial biopsies, which were provided by RWK and AJH. SGM, HW, HPT and TB conceived and supervised the project. They provided continuous conceptual input, designed the experiments and provided financial support. SB, NB and MF wrote the first draft of the manuscript, which was finalized by AJH, RWK, SGM, HW, HPT and TB.

## Supplementary Material

Additional file 1 **Supplementary Figure S1**. Quantitative K_2P_5.1 expression on the protein level in cells from RA patients compared to healthy controls. Western blot analysis of five individual RA patients (one to five) compared to two healthy controls (HD). Respective DAS28 scores are indicated at the upper part of the figure. Click here for file

Additional file 2 **Supplementary Figure S2**. Influence of therapeutic agents on K_2P_5.1 expression. **A) **Correlation between K_2P_5.1 expression levels (Δct values) on CD4^+ ^T lymphocytes and DAS28 scores is shown for patients with convential treatments (black diamonds), TNFα inhibitors (grey diamonds) and rituximab therapy (white diamonds). **B) **Patient subgroups are shown with conventional treatment (left side), TNFα inhibitors (middle side) and rituximab (right side). **C) **The left bar graph representation shows the correlation coefficients between DAS28 and K_2P_5.1 expression levels. The DAS28 score for the treatment subgroups is shown on the right side. Click here for file

## References

[B1] van Vollenhoven RF Treatment of rheumatoid arthritis: state of the art 2009 Nat Rev Rheumatol 2009 5 531 541 10.1038/nrrheum.2009.182 19798027

[B2] Feuchtenberger M Kneitz C Roll P Kleinert S Tony HP Sustained remission after combination therapy with rituximab and etanercept in two patients with rheumatoid arthritis after tnf failure: case report Open Rheumatol J 2009 3 9 13 10.2174/1874312900903010009 19479056 PMC2684711

[B3] van Gestel AM Haagsma CJ van Riel PL Validation of rheumatoid arthritis improvement criteria that include simplified joint counts Arthritis Rheum 1998 41 1845 1850 10.1002/1529-0131(199810)41:10<1845::AID-ART17>3.0.CO;2-K 9778226

[B4] Wells G Becker JC Teng J Dougados M Schiff M Smolen J Aletaha D van Riel PL Validation of the 28-joint Disease Activity Score (DAS28) and European League Against Rheumatism response criteria based on C-reactive protein against disease progression in patients with rheumatoid arthritis, and comparison with the DAS28 based on erythrocyte sedimentation rate Ann Rheum Dis 2009 68 954 960 10.1136/ard.2007.084459 18490431 PMC2674547

[B5] Bittner S Meuth SG Gobel K Melzer N Herrmann AM Simon OJ Weishaupt A Budde T Bayliss DA Bendszus M Wiendl H TASK1 modulates inflammation and neurodegeneration in autoimmune inflammation of the central nervous system Brain 2009 132 2501 2516 10.1093/brain/awp163 19570851 PMC3031313

[B6] Meuth SG Bittner S Meuth P Simon OJ Budde T Wiendl H TWIK-related acid-sensitive K+ channel 1 (TASK1) and TASK3 critically influence T lymphocyte effector functions J Biol Chem 2008 283 14559 14570 10.1074/jbc.M800637200 18375952

[B7] Bittner S Bobak N Herrmann AM Göbel K Meuth P Höhn KG Stenner MP Budde T Wiendl H Meuth SG Upregulation of K2P5.1 potassium channels in multiple sclerosis Annals of Neurology 2010 68 58 69 10.1002/ana.22010 20582984

[B8] Goronzy JJ Weyand CM Developments in the scientific understanding of rheumatoid arthritis Arthritis Res Ther 2009 11 249 19835638 10.1186/ar2758 PMC2787299

[B9] Maxwell L Singh JA Abatacept for rheumatoid arthritis Cochrane Database Syst Rev 2009 4 CD007277 19821401 10.1002/14651858.CD007277.pub2 PMC6464777

[B10] Dixon W Massey F Introduction to Statistical Analysis 1969 New York: McGraw-Hill Companies

[B11] Mima T Nishimoto N Clinical value of blocking IL-6 receptor Curr Opin Rheumatol 2009 21 224 230 10.1097/BOR.0b013e3283295fec 19365268

[B12] Funahashi K Koyano S Miura T Hagiwara T Okuda K Matsubara T Efficacy of tocilizumab and evaluation of clinical remission as determined by CDAI and MMP-3 level Mod Rheumatol 2009 19 507 512 10.1007/s10165-009-0203-z 19609487

[B13] Jones G Sebba A Gu J Lowenstein MB Calvo A Gomez-Reino JJ Siri DA Tomsic M Alecock E Woodworth T Genovese MC Comparison of tocilizumab monotherapy versus methotrexate monotherapy in patients with moderate to severe rheumatoid arthritis: the AMBITION study Ann Rheum Dis 2010 69 88 96 10.1136/ard.2008.105197 19297346 PMC3747519

[B14] Matsui T Komiya A Shimada K Nakayama H Tohma S Neutrophil CD64 as a marker of infection in patients treated with tocilizumab Mod Rheumatol 2009 19 696 697 10.1007/s10165-009-0223-8 19728012

