# Supplementary figures and images for: Expression of K2P5.1 potassium channels on CD4+ T lymphocytes correlates with disease activity in rheumatoid arthritis patients
Source: Arthritis Res Ther. 2011 Feb 11;13(1):R21. doi: 10.1186/ar3245 (PMC3241365; doi:10.1186/ar3245)

**A**

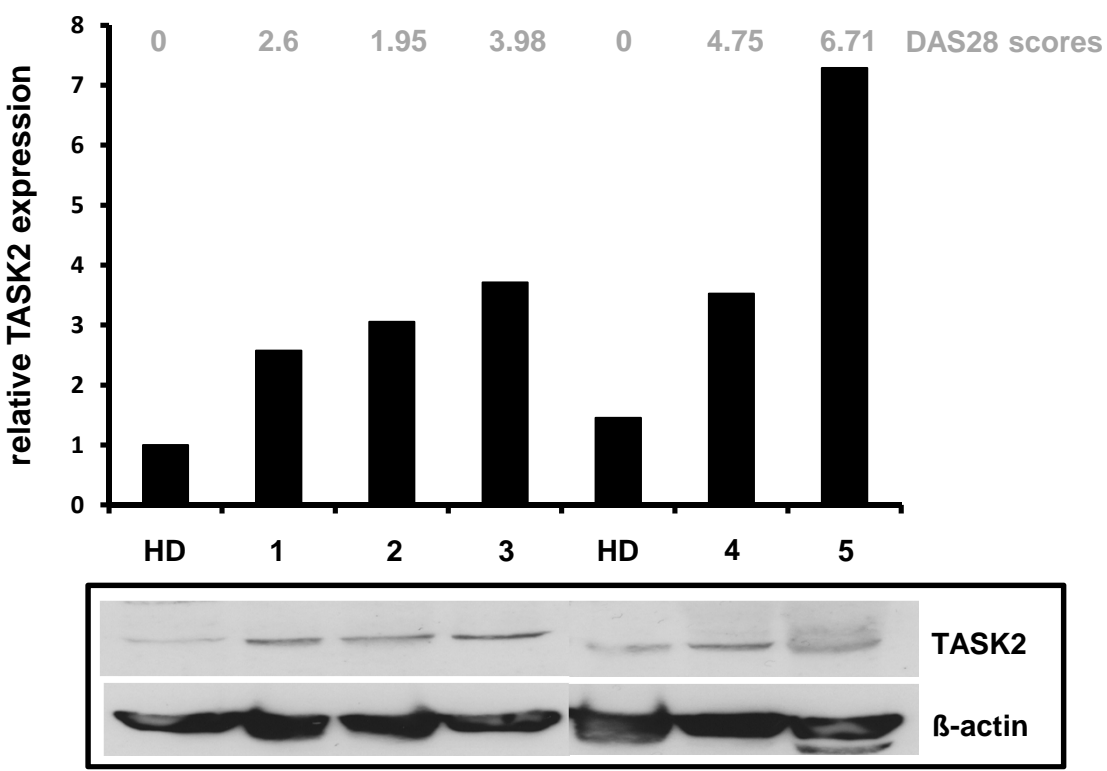

Supplement: Additional file 1 — Supplementary Figure S1. Quantitative K2P5.1 expression on the protein level in cells from RA patients compared to healthy controls. Western blot analysis of five individual RA patients (one to five) compared to two healthy controls (HD). Respective DAS28 scores are indicated at the upper part of the figure. [file ar3245-S1.PDF]

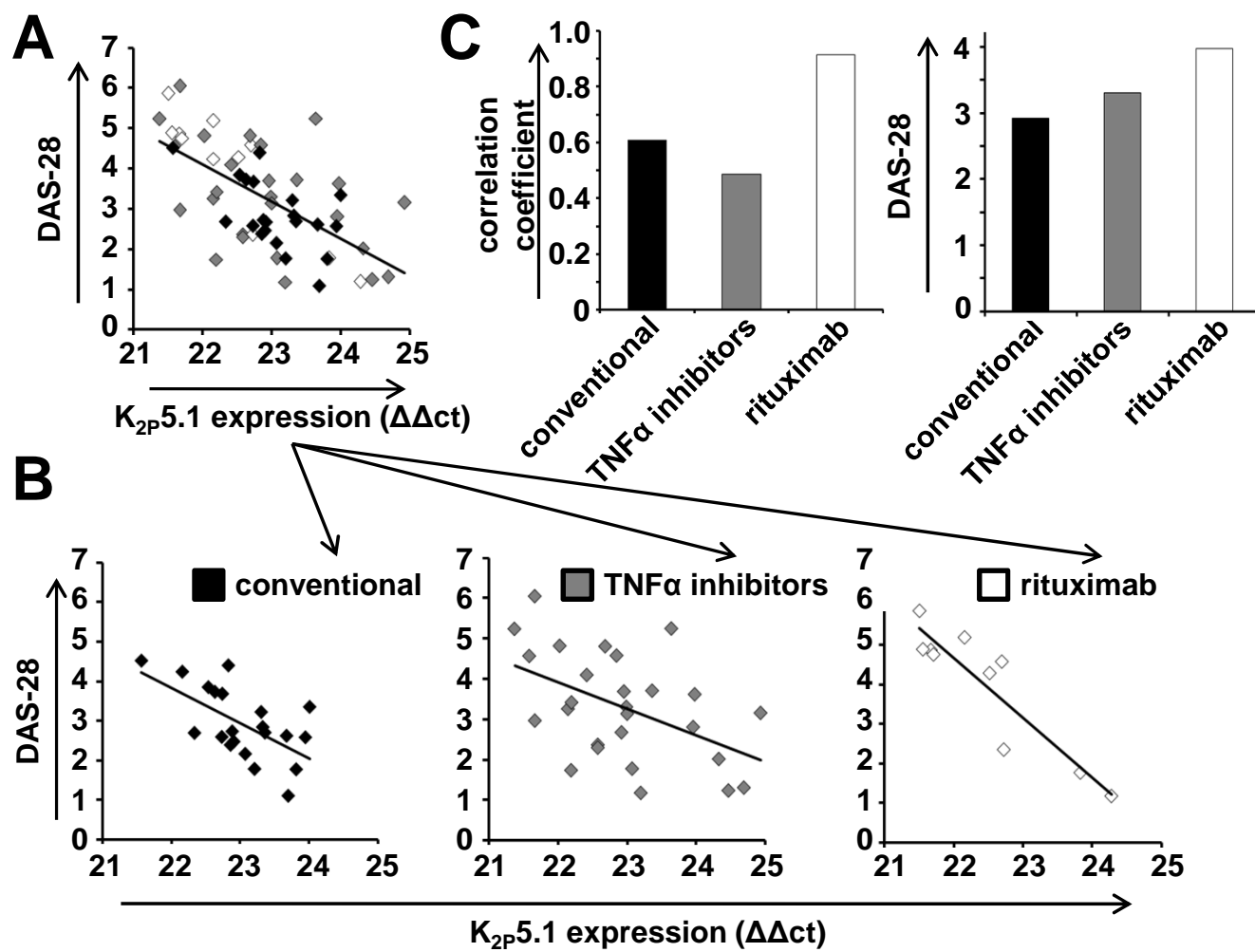

Supplement: Additional file 2 — Supplementary Figure S2. Influence of therapeutic agents on K2P5.1 expression. A) Correlation between K2P5.1 expression levels (Δct values) on CD4+ T lymphocytes and DAS28 scores is shown for patients with convential treatments (black diamonds), TNFα inhibitors (grey diamonds) and rituximab therapy (white diamonds). B) Patient subgroups are shown with conventional treatment (left side), TNFα inhibitors (middle side) and rituximab (right side). C) The left bar graph representation shows the correlation coefficients between DAS28 and K2P5.1 expression levels. The DAS28 score for the treatment subgroups is shown on the right side. [file ar3245-S2.PDF]
